# Knowledge, Attitudes, Motivations, and Practices of Blood Donation Among the Population of Saudi Arabia

**DOI:** 10.3390/healthcare14091143

**Published:** 2026-04-24

**Authors:** Saud Ibrahim Altilasi, Dima Hamze, Mazin Elsarrag, Muhammad Raihan Sajid, Salman Aldosari

**Affiliations:** 1 College of Medicine, Alfaisal University, Riyadh 11533, Saudi Arabia; 2 King Salman Hospital, Riyadh First Cluster, Ministry of Health, Riyadh 12769, Saudi Arabia; 3 Department of Pathology, College of Medicine, Alfaisal University, Riyadh 11533, Saudi Arabia

**Keywords:** blood donation, knowledge-attitude-practice (KAP), barriers, motivators, Saudi Arabia

## Abstract

**Background/Objectives**: Blood donation is a critical component of healthcare systems worldwide, yet donor recruitment remains challenging. This study evaluates the knowledge, attitudes, motivations, and practices (KAP) of blood donation among the general population in Saudi Arabia to identify key barriers and propose targeted interventions. **Methods**: A cross-sectional study was conducted using a structured, validated questionnaire distributed over five months (December 2022 to April 2023) via social media and in-person recruitment at the Central Blood Bank in Riyadh. A total of 1150 participants aged 18–60 years residing in Saudi Arabia were included in the final analysis. Statistical analysis was performed using SPSS version 22, with *p* < 0.05 considered significant. **Results**: Participants demonstrated moderate knowledge (mean score 5.43 ± 1.81 out of 9), with significantly higher scores among males, individuals aged 21–30 years, and those holding a bachelor’s degree. Attitudes toward donation were highly positive (mean score 15.46 ± 2.74 out of 20) and correlated with age, gender, marital status, and occupation. Despite this positive outlook, only 34.96% of participants had donated blood previously, although 95.25% expressed willingness to do so. Primary motivators included mobile donation units (89.22%) and paid leave (89.22%), whereas 51.22% of respondents considered current media campaigns ineffective. Common barriers to donation included health concerns (25.30%), time constraints (12.87%), and fear of needles (7.74%). **Conclusions**: This study reveals a critical disparity between positive public attitudes and actual donation practices in Saudi Arabia. To enhance donor participation, we recommend implementing convenient donation strategies such as mobile blood drives, workplace incentives, and more effective, culturally tailored educational campaigns. Addressing these factors could help Saudi Arabia improve its voluntary donation rates and ensure a sustainable, safe blood supply.

## 1. Introduction

Blood is an irreplaceable component of medical care, essential for managing emergencies, surgical procedures, and chronic conditions such as thalassemia and sickle cell anemia [1,2]. Unlike other medical resources, it cannot be artificially manufactured, making voluntary donor systems the cornerstone of sustainable blood supplies [3]. Globally, high-income countries maintain donation rates of 32.6 per 1000 population, while low- and middle-income countries (LMICs) average only 4.4–15.1 donations per 1000 [2]. Saudi Arabia’s donation rate of 13.8 per 1000 population [4] reflects progress but remains below the benchmarks set by high-resource nations, underscoring the need to address systemic and cultural barriers to donation.

A critical challenge is the reliance on replacement donors—individuals who donate only when a family member or friend requires transfusion [5]. In Saudi Arabia, an estimated 60% of donations fall into this category [6], perpetuating a reactive rather than proactive supply chain. Compounding this issue are persistent misconceptions regarding the safety of the donation process. While attitudes are generally positive, earlier studies indicated that a significant minority (e.g., 50% in a study by Baig et al., 2013) perceived blood donation as risky, though more recent research suggests such misconceptions may be decreasing [7,8]. Such attitudes are exacerbated by limited public awareness campaigns and logistical hurdles, such as the inaccessibility of donor centers [9].

The demand for blood in Saudi Arabia is further amplified by high rates of road traffic accidents (RTAs), which account for 80% of trauma admissions and cost the economy 21 billion USD annually [10]. Concurrently, obstetric hemorrhages and chronic transfusion-dependent conditions strain existing supplies [11,12]. While the Saudi Ministry of Health has expanded infrastructure—establishing 140 donation centers and conducting awareness campaigns [9]—studies reveal persistent knowledge gaps, particularly among women, younger demographics, and those with lower educational attainment [13]. Mobile donation units and workplace incentives could address these gaps, yet such strategies remain underutilized.

This study provides a comprehensive analysis of knowledge, attitudes, motivations, and practices (KAP) related to blood donation across Saudi Arabia’s sociodemographic groups. It aims to identify key barriers and facilitators, and to analyze the sociodemographic factors associated with these outcomes. By identifying gaps and target populations, this study aims to inform evidence-based policies that bolster voluntary donation rates. The findings will guide efforts to align Saudi Arabia’s blood supply system with WHO recommendations, ensuring resilience against rising clinical demands.

## 2. Methodology

### 2.1. Study Design and Setting

This prospective cross-sectional study was conducted among the general population of Saudi Arabia over a five-month period from December 2022 to April 2023. The study aimed to evaluate knowledge, attitudes, practices, and motivations regarding blood donation across different demographic groups.

### 2.2. Participant Selection

The study included male and female individuals aged between 18 and 60 years residing in Saudi Arabia. This age criterion was selected to align with standard blood donor eligibility guidelines, defining the adult population most likely to be potential donors.

### 2.3. Sample Size Determination

The minimum required sample size was calculated as 384 participants using an online sample size calculator (https://www.surveysystem.com/), with a 95% confidence level and 5% margin of error. This calculation was based on Saudi Arabia’s estimated population of 34 million according to the latest census data.

### 2.4. Data Collection Procedures

Data were collected using a validated, self-administered questionnaire available in both Arabic and English versions. The questionnaire was originally developed by Alfouzan (2014) and permission for its use was obtained from the authors through professional correspondence [14]. The instrument contained six distinct sections assessing demographic characteristics, knowledge about blood donation, attitudes toward donation, self-reported donation practices, barriers to donation, and motivational factors.

The questionnaire was distributed through two primary methods: digital dissemination via social media platforms including Twitter and WhatsApp, and in-person recruitment at the Central Blood Bank in Riyadh along with various public locations. For in-person recruitment, participants accessed the questionnaire by scanning a QR code displayed at these locations. All responses were collected anonymously through Google Forms, with no personal identifiers recorded to ensure participant confidentiality.

### 2.5. Ethical Considerations

The study protocol received ethical approval from the Institutional Review Board at King Saud Medical City (Approval number: H1R1-06-Nov22-01) prior to commencement. The research adhered to all relevant ethical guidelines for human subjects research.

### 2.6. Data Analysis

Statistical analysis was performed using SPSS version 22 (IBM Corp., Armonk, NY, USA). Continuous variables were expressed as mean ± standard deviation, while categorical variables were presented as percentages. The normality of continuous variable distributions was assessed using the Kolmogorov–Smirnov test. For comparisons of non-normally distributed continuous variables, the Mann–Whitney U test or Kruskal–Wallis test was employed as appropriate. Categorical variables were analyzed using either the Chi-square test or Fisher’s exact test. A *p*-value of less than 0.05 was considered statistically significant for all analyses.

## 3. Results

### 3.1. Participant Characteristics

A total of 1150 individuals participated in this study. As shown in Table 1, nearly half (47.1%) were aged 31–50 years, with a female predominance (65.7%). Most participants were Saudi nationals (87.4%), married (52.3%), and held at least a bachelor’s degree (77.1%).

### 3.2. Knowledge About Blood Donation

Participants demonstrated moderate knowledge with a mean score of 5.43 ± 1.81 out of 9 (Table 2). While most (99.5%) knew their blood group, which is commonly recorded on driving licenses in Saudi Arabia, only 54.3% identified the minimum donation age, and 35.1% knew the recommended interval between donations.

Knowledge levels varied significantly by demographics (Table 3). The 21–30 age group scored highest (5.60 ± 1.86, *p* = 0.009), as did males (5.61 ± 1.79 vs. 5.33 ± 1.81, *p* = 0.010) and bachelor’s degree holders (5.57 ± 1.78, *p* < 0.001).

### 3.3. Attitudes Toward Donation

Participants showed positive attitudes (mean 15.46 ± 2.74 out of 20, Table 4). Males (16.06 ± 2.71), married individuals (15.82 ± 2.70), and civil sector workers (16.24 ± 2.62) had significantly more favorable attitudes (all *p* < 0.001).

### 3.4. Donation Practices

Only 34.96% had donated blood previously (Table 5). Males were significantly more likely to have donated than females (69.37% vs. 16.95%, *p* < 0.001). Most donors (95.75%) reported positive experiences, and 95.25% would donate again.

Donation practices varied significantly by demographic factors. The proportion of participants who had ever donated blood increased with age, from 11.22% among those aged 18–20 years to 46.97% among those aged 51–60 years (*p* < 0.001). Similarly, donation rates were higher among participants with higher educational attainment, with 47.31% of those holding a higher degree (master’s, PhD) having donated, compared to 26.23% of those with a high school education (*p* < 0.001)

### 3.5. Barriers to Donation

**Figure 1:**
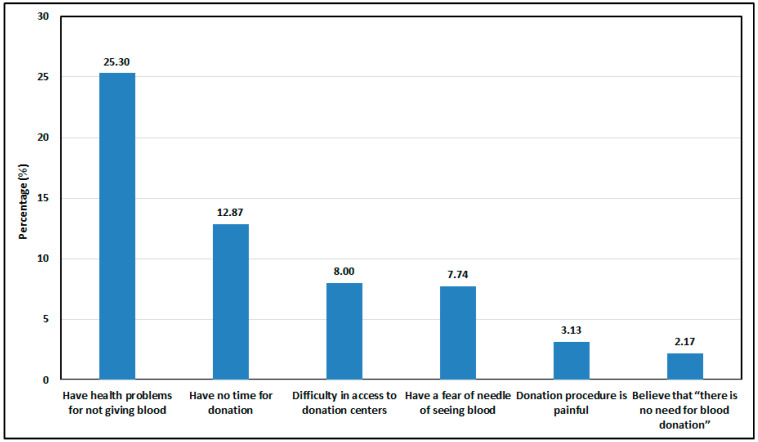
Reasons for not donating blood.

As illustrated in Figure 1, primary barriers included health issues (25.3%), time constraints (12.87%), and needle fear (7.74%).

### 3.6. Motivational Factors

Most participants endorsed mobile units (89.22%) and paid leave (89.22%) as effective motivators (Table 6). While 48.78% found media campaigns encouraging, 51.22% disagreed.

## 4. Discussion

Recruiting sufficient and safe blood donors in Saudi Arabia presents a persistent and multifaceted challenge, particularly in light of rapid population growth and healthcare expansion. Ensuring an adequate blood supply depends not only on the quantity of donations but also on public awareness, positive attitudes, and consistent voluntary participation [15]. This study aimed to assess the knowledge, attitudes, practices, and motivations regarding blood donation among the Saudi population, while identifying barriers and proposing improvement strategies.

The study sample included 1150 participants, primarily aged 31–50 years, providing a broader demographic representation than Beyene’s study [16], which focused on individuals aged 26–35 years. The inclusion of a wider age range enhances the reliability and generalizability of the findings, as blood donation behaviors often vary across life stages. Females represented 65.7% of the sample, consistent with Gunjaliya et al. [17], reflecting increasing female engagement in health-related research despite traditionally lower donation rates. Most participants (60.9%) held bachelor’s degrees, indicating a population with high educational attainment—an attribute previously associated with improved health literacy and awareness of blood donation processes [17].

Despite this favorable demographic profile, knowledge gaps persisted, particularly regarding donor eligibility, safe donation intervals, and misconceptions about health effects. Although participants demonstrated acceptable knowledge levels—similar to findings from Alzaben et al. [18] and Kowsalya et al. [19]—other studies, such as Giri and Phalke [20], Omaish et al. [21], Aslami et al. [22], and Wiwanitkit [23], reported higher knowledge levels. Such variations may stem from differences in population demographics, cultural contexts, and study design. Interestingly, despite widespread access to modern communication tools, gaps in accurate understanding remain, suggesting that targeted public health education is still needed to enhance knowledge consistency.

Knowledge levels were significantly associated with male gender, higher education, older age, and civil employment, corroborating Ethiopian findings that education, sex, and age predict blood donation practices [24]. Similarly, Elnajeh et al. [25] and Singh and Bhatt [26] reported that males tend to have greater awareness and experience regarding blood donation. Moreover, 78.1% of participants acknowledged the importance of pre-donation testing, aligning with Salem et al. [27], who emphasized the role of screening, particularly for HIV, in ensuring blood safety. Elnajeh et al. [25] also documented high knowledge levels (97.1%), with education serving as a significant predictor.

Participants demonstrated overall positive attitudes toward blood donation, consistent with Tsega et al. [24], Giri and Phalke [20], and Wiwanitkit [23]. Male gender, older age, marriage, and employment were associated with more favorable attitudes. Al-Drees [28] reported that Saudi men are more likely to donate blood, while Nigatu and Demissie [29] observed that women had higher awareness but lower donation rates, suggesting sociocultural factors influence practice. In the Saudi context, these barriers may be compounded by cultural norms related to gender. For instance, women may face restrictions on independent mobility, limiting their ability to travel to donation centers, or may have concerns about modesty in mixed-gender clinical settings. To overcome these barriers, targeted strategies such as women-only donation sessions, mobile donation units stationed at female-only spaces (e.g., universities, shopping centers), and culturally tailored awareness campaigns that address specific concerns of female donors could be implemented. The current findings are in agreement with Al-Haqqan et al. [30] regarding gender’s predictive value but contrast with Singh et al. [26], who found more favorable female attitudes. The positive attitudes documented across Saudi, Pakistani, and Indian populations [7,31] suggest widespread willingness to donate, though attitudinal positivity alone may not always translate into consistent donor behavior.

The voluntary donation rate in this study was 57%, though only 35.1% of respondents knew the correct donation intervals. Encouragingly, 95.25% expressed willingness to donate again, contrasting sharply with Alsarafandi et al. [32], where 83.3% had never donated and only 12.5% intended to donate in the future. These results parallel those of Lee and Muthalib [33], indicating that prior donors are more likely to donate again. However, earlier research [34,35] attributed low overall donation rates to misinformation, fear, and negative attitudes, underscoring the need for sustained education and reassurance.

The most frequently reported barriers included health issues (25.30%), time constraints (12.87%), limited access (8%), fear of needles (7.74%), pain misconceptions (3.13%), and lack of awareness (2.17%). These findings mirror Alsalmi et al. [7], although fear-related factors appeared less prominent in this study, possibly indicating cultural normalization of medical procedures. Still, logistical barriers such as limited time and accessibility remain significant, particularly among working adults. Voluntary donations (57%) outweighed replacement donations (15.5%), consistent with regional trends emphasizing social duty as a primary motivator [3]. Nonetheless, overreliance on replacement donations risks compromising both supply stability and transfusion safety [12]. Future studies should specifically investigate the rate at which replacement donors convert to regular voluntary donors, as this represents a key opportunity for stabilizing the blood supply. Experiences from successful systems in Europe and North America demonstrate that continuous donor retention programs and targeted educational efforts can enhance the sustainability of voluntary blood donation networks [36].

Participants strongly supported implementing mobile blood donation units (89.22%) and providing paid leave for donors (89.22%). These findings highlight convenience and recognition as important motivational factors. However, only 48.78% of respondents believed current media campaigns were effective, suggesting opportunities for improvement. Previous evidence indicates that first-time donors respond best to personal appeals and traditional media [37,38], while social media has emerged as a powerful platform for promoting health-related prosocial behaviors [39]. Thus, integrating culturally sensitive digital campaigns, influencer collaborations, and community-driven narratives could significantly enhance donor recruitment and retention across Saudi Arabia.

These findings have significant practical implications for public policy and national blood donation programs. The Ministry of Health could operationalize the strong public support for mobile units by establishing a regular schedule of mobile blood drives at high-traffic locations such as malls, universities, and public squares, particularly in underserved areas. The overwhelming endorsement of paid leave as a motivator suggests that policy changes requiring employers to provide paid time off for blood donation could substantially increase participation. Furthermore, the low perceived effectiveness of current media campaigns indicates a need for a strategic overhaul. Campaigns should be segmented to target specific demographics identified in this study as having lower knowledge and donation rates, such as women, younger individuals, and those with lower educational attainment. Leveraging social media platforms and collaborating with respected community influencers could enhance campaign reach and credibility. These policy recommendations align with the Saudi Ministry of Health’s strategic goal of achieving a safe, sustainable, and voluntary blood supply.

In conclusion, this study demonstrates that while the Saudi population exhibits generally positive attitudes and moderate knowledge about blood donation, substantial gaps persist in practice and perception. Strengthening educational outreach, improving donation accessibility, and leveraging both traditional and digital media are essential for fostering a sustainable, safe, and fully voluntary blood donation culture in the Kingdom. Future national strategies should prioritize long-term awareness initiatives, workplace-based donation programs, and continuous evaluation of public engagement efforts to ensure the adequacy and safety of the national blood supply.

## 5. Limitations

The study is based on self-reported answers, which may include recollection bias or social desirability bias, causing participants to underestimate or overestimate their knowledge, attitudes, and habits toward blood donation. Furthermore, the cross-sectional design precludes any inference of causality. The use of convenience sampling via social media and in-person QR codes did not allow for the calculation of a response rate, and the sample may be subject to selection bias. Participants recruited at the Central Blood Bank may have had a higher baseline interest in blood donation, potentially biasing attitudes and practices upward. Additionally, individuals who chose to respond to an online survey may have systematically differed from the general population in terms of health literacy or engagement with health topics. The sample also overrepresents women and highly educated individuals, which limits the generalizability of the findings to the entire Saudi population. These factors collectively restrict the extent to which the study’s findings can be generalized to the broader population.

## 6. Conclusions

This study revealed a critical disparity between participants’ positive attitudes toward blood donation and their insufficient knowledge about the process. While respondents demonstrated highly favorable views, their understanding of key donation requirements remained inadequate. The analysis identified significant associations between knowledge levels and demographic factors including age, gender, education, and occupation. Similarly, attitudes showed meaningful correlations with age, gender, marital status, and occupational background. Despite these positive dispositions, actual donation practices remained limited, though encouragingly, most donations occurred voluntarily. A strong willingness to donate was evident among participants, with many actively encouraging family members to participate. Mobile donation units emerged as the most effective motivator according to participant feedback, while nearly half expressed skepticism about media campaigns’ ability to promote blood donation effectively. These findings highlight both the potential for increasing donation rates through convenient access points like mobile units and the need for more impactful public education strategies to address persistent knowledge gaps and optimize donor recruitment efforts.

## Figures and Tables

**Table 1:** Demographic characteristics of the participants (N = 1150).

		Number	%
Age	18–20	98	8.5
	21–30	444	38.6
	31–50	542	47.1
	51–60	66	5.7
Gender	Male	395	34.3
	Female	755	65.7
Nationality	Saudi	1005	87.4
	Non-Saudi	145	12.6
Marital Status	Single	548	47.7
	Married	602	52.3
Level of education	Below high school	20	1.7
	High school	244	21.2
	Bachelor’s degree	700	60.9
	Higher degree (master, PhD)	186	16.2
Occupation	Student	269	23.4
	Civil	314	27.3
	Private	279	24.3
	Military sector	30	2.6
	Unemployed	258	22.4

**Table 2:** Assessment of correct answers about blood donation knowledge.

	Right Answers	
	Number	%
What is your blood group?	1144	99.5
What is the blood type that can donate to any individual?	821	71.4
What is the minimum age for blood donation?	625	54.3
What is the minimum weight for blood donation?	635	55.2
What is the minimum interval between two blood donations?	404	35.1
Do you know where the location of the blood bank in our community is?	831	72.3
Do you think that donating blood can cause infectious diseases, such as HIV, during the process of donating?	604	52.5
Can diabetic or hypertensive patients donate blood?	279	24.3
Does the blood bank do tests on donated blood for infections like HIV, Hepatitis B and C to ensure safe blood transfusion?	898	78.1

**Table 3:** Association between demographic characteristics and knowledge scores.

		Mean	SD	*p* Value
Age	18–20	5.00	1.58	0.009 *
	21–30	5.60	1.86	
	31–50	5.37	1.81	
	51–60	5.35	1.54	
Gender	Male	5.61	1.79	0.010 *
	Female	5.33	1.81	
Nationality	Saudi	5.40	1.81	0.288
	Non-Saudi	5.59	1.78	
Marital Status	Single	5.45	1.76	0.884
	Married	5.41	1.84	
Level of education	Below high school	4.85	1.79	<0.001 *
	High school	4.98	1.86	
	Bachelor’s degree	5.57	1.78	
	Higher degree (master, PhD)	5.53	1.75	
Occupation	Student	5.40	1.75	0.002 *
	Civil	5.77	1.76	
	Private	5.58	1.70	
	Military sector	4.50	2.19	
	Unemployed	4.98	1.87	
Overall knowledge (out of 9)		5.43	1.81	

Note: Asterisks (*) indicate statistically significant differences (*p* < 0.05).

**Table 4:** Association between demographics and attitude scores.

		Mean	SD	*p* Value
Age	18–20	14.57	2.62	<0.001 *
	21–30	15.11	2.76	
	31–50	15.80	2.67	
	51–60	16.27	2.69	
Gender	Male	16.06	2.71	<0.001 *
	Female	15.15	2.70	
Nationality	Saudi	15.47	2.73	0.775
	Non-Saudi	15.35	2.79	
Marital Status	Single	15.07	2.72	<0.001 *
	Married	15.82	2.70	
Level of education	Below high school	15.25	2.49	0.137
	High school	15.19	2.62	
	Bachelor’s degree	15.44	2.78	
	Higher degree (master′s, PhD)	15.89	2.72	
Occupation	Student	14.79	2.63	<0.001 *
	Civil	16.24	2.62	
	Private	15.65	2.75	
	Military sector	15.70	2.76	
	Unemployed	14.98	2.72	
Overall attitude (out of 20)		15.46	2.74	

Note: Asterisks (*) indicate statistically significant differences (*p* < 0.05).

**Table 5:** Blood donation practices among participants.

		Number	%
Have you donated blood before?	Yes	402	34.96
	No	748	65.04
How many times have you donated blood in your life?	Once	128	31.84
	2 to 5	162	40.30
	Several times	91	22.64
	Every year	21	5.22
What was the reason for the previous donation?	Voluntary	228	57.00
	For relatives and friends	62	15.50
	Both of them	102	25.50
	Other	8	2.00
How was your blood donation experience?	Positive	383	95.75
	Negative	17	4.25
Will you donate blood again if asked?	Yes	381	95.25
	No	19	4.75
Do you encourage your relatives and friends to donate blood?	Yes	386	96.98
	No	12	3.02

**Table 6:** Motivations for blood donation.

		Number	%
Do you think that the media encourages people to donate blood?	Yes	561	48.78
	No	589	51.22
Do you think that mobile donation caravans in public areas (malls, plazas, streets) are a good motivational factor for donating blood?	Yes	1026	89.22
	No	124	10.78
Do you think that one day off from work is a motivational factor for donation?	Yes	1026	89.22
	No	124	10.78
Which of these motivations is suitable for donation:	Money	392	34.09
	Gifts	352	30.61
	Others	406	35.30

## Data Availability

The original contributions presented in this study are included in the article/Supplementary Materials. Further inquiries can be directed to the corresponding author.

## Supplementary Materials

The following supporting information can be downloaded at: https://www.mdpi.com/article/10.3390/healthcare14091143/s1.
